# *Helicobacter pylori* from Peptic Ulcer Patients in Uganda Is Highly Resistant to Clarithromycin and Fluoroquinolones: Results of the GenoType HelicoDR Test Directly Applied on Stool

**DOI:** 10.1155/2017/5430723

**Published:** 2017-05-07

**Authors:** Denish Calmax Angol, Ponsiano Ocama, Tess Ayazika Kirabo, Alfred Okeng, Irene Najjingo, Freddie Bwanga

**Affiliations:** ^1^Department of Medical Microbiology, Makerere University College of Health Sciences, P.O. Box 7072, Kampala, Uganda; ^2^Department of Midwifery, Faculty of Health Sciences, Lira University, P.O. Box 1035, Lira, Uganda; ^3^Department of Medicine, Makerere University College of Health Sciences, P.O. Box 7072, Kampala, Uganda; ^4^MBN Clinical Laboratories, Plot 28 Nakasero Road, Kampala, Uganda

## Abstract

**Background:**

Around 70–90% of peptic ulcer disease (PUD) is due to* Helicobacter pylori* and requires treatment with antimicrobials to which these bacteria are susceptible. Common* H. pylori* diagnostic tests do not provide drug susceptibility data. Using the GenoType HelicoDR PCR test designed for gastric biopsies for simultaneous detection of* H. pylori* and its resistance to clarithromycin (CLA)/fluoroquinolones (FLQ), we present evidence for stool as an optional test specimen and also provide data on prevalence of* H. pylori* resistance to CLA and FLQ in Uganda.

**Methods:**

Stool from 142 symptomatic PUD patients at three hospitals in Kampala was screened for* H. pylori* using a rapid antigen test. The GenoType HelicoDR test was run on all* H. pylori* antigen positives to determine PCR positivity and resistance to CLA/FLQ.

**Results:**

Thirty-one samples (22%) were* H. pylori* antigen positive, and 21 (68%) of these were* H. pylori* PCR positive. Six of the 21 (29%) were resistant to CLA and eight to FLQ (42%), while two gave invalid FLQ resistance results.

**Conclusion:**

Stool is a possible specimen for the GenoType HelicoDR test for rapid detection of* H. pylori* and drug resistance. In Uganda,* Helicobacter pylori* is highly resistant to CLA and FLQ.

## 1. Introduction

*Helicobacter pylori* is a motile Gram-negative, oxidase-, catalase-, and urease-positive, and microaerophilic bacillus [1]. Being microaerophilic, it needs only about 4% oxygen, 5% carbon dioxide, and 5% hydrogen for its growth and survival. This bacterium has been reported to be the cause of 90% of duodenal and 70% of gastric ulcer cases and may lead to stomach and/or duodenal perforation and fatal bleeding, and it is also associated with gastric cancer [2–4]. It is estimated that half of the world's population is infected with* H. pylori*, making it the most widespread infection in the world [5–7]. The actual burden of infection varies from nation to another. In Western Europe, North America, Australia, and parts of Asia, the proportion of infected people is estimated to be about 25% [7]. Developing countries have much higher infection rates, and in Africa, it is estimated that 61–100% of the population is infected and at risk of PUD and the other associated diseases [2, 8–11].

Effective management of PUD caused by* H. pylori* requires a combination of antimicrobials and proton pump inhibitors (PPIs, which inhibit secretion of acid by the stomach). The antimicrobial agents commonly used in first-line treatment include clarithromycin (CLA), metronidazole, and amoxicillin, while others such as fluoroquinolones (FLQ) and tetracycline are used in second-line regimens. A number of studies have shown that* H. pylori* has developed resistance to the most commonly used antimicrobial agents used in PUD treatment. Work reported by Lee et al. from 2003 to 2012 found that resistance of* H. pylori* to CLA increased from 17 to 24% while for the FLQ (levofloxacin and moxifloxacin) from 5% to 28% and amoxicillin from 6% to 15% [12]. Another study by Kumala and Rani in 2006 found that* H. pylori* resistance to CLA was 28%, to amoxicillin at 19%, and to FLQ (ciprofloxacin, norfloxacin, and ofloxacin at 7%, sparfloxacin and gatifloxacin at 3%, and levofloxacin and moxifloxacin at 1%) [13]. Similarly, the prevalence of resistance in 128* H. pylori* strains isolated in 2004-2005 to FLQ (ciprofloxacin MIC > 1 mg/L) was 17% (22 strains) [14], while for CLA the pretreatment and posttreatment prevalence of resistance were 19% (23/123) and 69% (9/13), respectively [15]. This CLA resistance was relatively high compared to that reported as the national prevalence of resistance in France, which stood at 13% [16]. Results of a study of 2204 patients in Europe showed that* H. pylori* resistance rates were 16% to CLA, 14% to levofloxacin, and 35% to metronidazole. Resistance was significantly higher for CLA and levofloxacin in Western, Central, and Southern Europe (>20%) than in Northern European countries (<10%) [17]. Most of these studies recommended that in addition to accurate detection of* H. Pylori*, susceptibility tests (resistance diagnosis) should be done prior to treatment.

The diagnosis of* H. pylori* infection is currently carried out using four major methods [18, 19]:* (i) breath test*, in which the patient drinks ^13^C- or ^14^C-labelled urea, which the bacterium metabolizes producing labelled carbon dioxide that can be detected in the breath (the test is quick in the initial detection of the bacteria but requires complex equipment and cannot be used to detect drug resistance);* (ii) stool antigen test,* which detects* H. pylori* in the faeces for the purpose of initial detection of the bacteria and recurrences after antibiotic therapy (just like breath test, the* H. pylori* stool antigen test cannot detect drug resistance);* (iii) blood antibody test,* which detects antibodies to* H. pylori* in blood samples for purpose of detecting prior exposure but cannot also detect drug resistance; and* (iv) gastric biopsy examination test* (for culture, rapid urease test, or histological examination), which confirms* H. pylori* infection in PUD cases. These test approaches have before been regarded as reference tests but they are invasive, expensive, and not easily adopted in routine diagnostics in resource-poor settings. Culture and phenotypic antimicrobial susceptibility testing of* H. pylori* requires up to 10–14 days, on expensive special media and special microaerophilic environments, and it is hardly performed in routine clinical laboratories in the resource-poor settings [20].

The GenoType HelicoDR test (GTHDT,* Hain Lifescience Nehren, Germany*) was developed recently. This is a multiplex PCR-based assay for simultaneous detection of* H. pylori* and its resistance to two-key antibiotics, CLA and FLQ [21]. The test was originally designed for use on gastric biopsy or culture materials to detect mutations in* gyrA* gene codons 87 and 91 for resistance to FLQ and in the 23S rRNA genes for resistance to CLA. The test involves DNA extraction, amplification, and solid-phase reverse hybridization onto probes fixed on a nitrocellulose membrane. Several studies evaluating the GTHDT for diagnosis of CLA/FLQ-resistant* H. pylori* have been conducted on biopsy specimens or cultured isolates, but as a diagnostic test for* H. pylori* performed directly on stool specimens the data is limited. In 2009, Cambau et al. evaluated the GTHDT using gastric biopsy and clinical strains, and the sensitivity and specificity for detecting resistance were high, that is, 94% and 99% for CLA and 87% and 98.5% for FLQ, respectively [22].

However, the requirement of stomach or duodenal biopsy specimen is a major limitation to use of this test in poor countries where endoscopic facilities as well as the other tests remain extremely scarce and expensive. Obtaining these biopsy specimens involves an invasive and expensive endoscopic procedure not usually acceptable by patients. Consequently, this potentially useful test has attracted little use in routine patient care, particularly in the developing countries where* H. pylori* infection is highest, yet resources for endoscopy are very limited. Hence, very few data on* H. pylori* resistance have come from these resource-poor countries. An alternative noninvasive, less costly, easy to collect specimen such as stool, which is also more acceptable to patients, would be needed for use with the GTHDT in routine PUD patient care particularly in the detection of* H. pylori* and diagnosis of drug resistance. This study was undertaken to determine the performance of the GTHDT on stool as a starting material for molecular diagnosis of* H. pylori* and its resistance to CLA and FLQ simultaneously and determine the prevalence of* H. pylori* resistance to CLA/FLQ in Uganda.

## 2. Materials and Methods

### 2.1. Study Design and Approval

This was a cross-sectional study carried out from November 2012 to July 2013. This study was approved by the School of Biomedical Sciences Research and Ethics Committee (SBS-REC) of Makerere University College of Health Science (SBS 076) and by the Mulago Hospital Research and Ethics Committee (MREC: 374).

### 2.2. Study Site and Setting

The study was conducted at three sites (Mulago, Mengo, and Case Medical Centre Hospitals) in Kampala, Uganda. The Mulago National Referral Hospital has a gastrointestinal (GI) unit with both inpatient and outpatient services. The outpatient service runs weekly with about 40 patients seen every week, about one-half due to either established or suspected PUD cases, some of which get admitted into the wards. Mengo Hospital and Case Medical Centre are tertiary referral Nongovernmental Organisation and private hospitals within Kampala City, respectively. They provide care for many patients with various ailments including gastroenterological cases. Recruitment of the study participants and sample collections were done at these three hospitals. All laboratory assays were performed at MBN Clinical Laboratories, Kampala, Uganda, which offer advanced medical laboratory diagnostic services for patient care and research.

### 2.3. Study Population and Inclusion Criteria

The study included patients with suspected PUD who were at least 13 years old attending the GIT clinics at the study hospitals. Inclusion was based on having symptoms and signs of PUD as judged by the attending physician and consent to participate in the study. These symptoms included burning pain in the epigastrium with or without any of the following: bloating, nausea, dark or black stool, vomiting blood, or weight loss. The patients were individually approached to obtain informed consent (or assent with legal guardian consent) and consecutively recruited into the study. A predesigned case report form was used to collect both demographic and clinical data.

### 2.4. Sample Collection

Upon consenting, each participant was provided with a sterile dry stool container and instructed to collect three scoopful of the stool into the stool container. The stool specimen was then placed in a cool box and transported to the laboratory within 2 hours of sample collection.

### 2.5. Laboratory Methods

#### 2.5.1. *H. pylori* Stool Antigen Test

All stool samples were tested for* H. pylori* antigen using an immunochromatopgraphy assay, the Taytec* H. pylori* stool antigen rapid diagnostic kit (Taytec Enterprises Canada) whose manufacturer's claimed sensitivity and specificity were above 95% [23, 24]. In the assay, the* H. pylori* antigen in stool reacts with conjugated-red latex particles sensitised with anti-*H. pylori* monoclonal antibodies. The* H. pylori*-conjugate complex migrates along the membrane by capillarity and binds to the specific antibody molecules fixed at the reaction zone. The excess of the complex keeps migrating through the membrane until reaching the control (C) zone where it binds to another specific antibody coated to the membrane forming a green band. Presence of the green band confirms the functionality of the test. In the procedure, about 50 mg of each stool sample was picked using the applicator stick and emulsified in the buffer and four drops of the emulsion were dispensed into the well of the cassette. The interpretations of the results (bands) for the* H. pylori* stool antigen test were as follows: a positive result was indicated by two bands (control = green and test = red), negative by only the control band (green), and invalid by either only red or no band [24]. The detailed procedure is found in the Taytec, Aldercrest Dr. Mississauga, Canada,* H. pylori* stool antigen test kit insert [24].

#### 2.5.2. GTHDT

Only* H. pylori* antigen positive stool samples were subjected to this test.

*DNA Extraction from Stool*. This was performed using the QIAamp® DNA Stool Mini Kit (Qiagen, Hilden Germany, Catalogue Number: 51504). The procedures are detailed in the QIAamp DNA Stool Handbook [25]. Briefly, 180 to 220 mg of each* H. pylori* antigen positive stool specimen was transferred to 1.5 mL microcentrifuge tube and the ASL buffer was added to lyse the bacterial cells. The suspension was then heated for 5 min at 70°C and then vortexed and centrifuged to pellet debris. The supernatant was then transferred into a new 1.5 mL microcentrifuge followed by addition of the InhibitEX tablet and incubated at 65°C for 5 minutes to allow inhibitors to be adsorbed to the InhibitEX matrix. After centrifugation, 200 *μ*L of the resultant supernatant was transferred to 15 *μ*L proteinase K in another 1.5 mL microcentrifuge tube. Then 200 *μ*L of absolute ethanol was added to the lysate to purify the DNA. This was then washed with 500 *μ*L of buffer AW1 followed by the same amount of buffer AW2. It was then eluted using 50 *μ*L of elution buffer AE after incubation at 65°C for 10 minutes. Eight microlitres of the pure DNA was added to 45 *μ*L PCR reagent mix ready for amplification.

*Multiplex PCR Amplification of Extracted DNA*. As recommended by the manufacturer, the composition of each reaction mix was 35 *μ*L of Primer Nucleotide Mix (PNM, containing biotin labelled primer sequences), 5 *μ*L of 10x PCR buffer, 5 *μ*L of 25 mM MgCl_2_, 0.2 *μ*L of HotStarTaq polymerase (1 U), and 8 *μ*L of the extracted DNA solutions. The PCR reaction tubes were loaded and amplified on the GTQ Cycler 96 (Hain LifeScience Nehren, Germany). The amplification program included phase 1, one cycle at 95°C for 15 min; phase 2, 10 cycles at 95°C for 30 s and at 58°C for 2 min; phase 3, 25 cycles at 95°C for 30 s, 53°C for 40 s, and 70°C for 40 s; and phase 4, one cycle at 70°C for 8 min. Details of the PCR reagent recipe, primer sequences, what they detect, and detailed procedure are found in the GTHDT kit insert, Lot number JF0045 [21].

*Detection of Amplification Products*.* This was performed using a solid-phase reverse hybridization technique* on the TwinCubator according to the manufacturer's instructions outlined in the GTHDT kit insert [21]. The hybridization process entailed chemical denaturation of double stranded DNA at room temperature for 5 min, hybridization of single stranded DNA with complementary DNA probes fixed on a nitrocellulose membrane strip in the hybridization buffer (HYB, green) at 45°C for 30 min, stringent washing, conjugation at room temperature for 30 min, and enzymatic detection of the reaction products [21].

**Figure 1:**
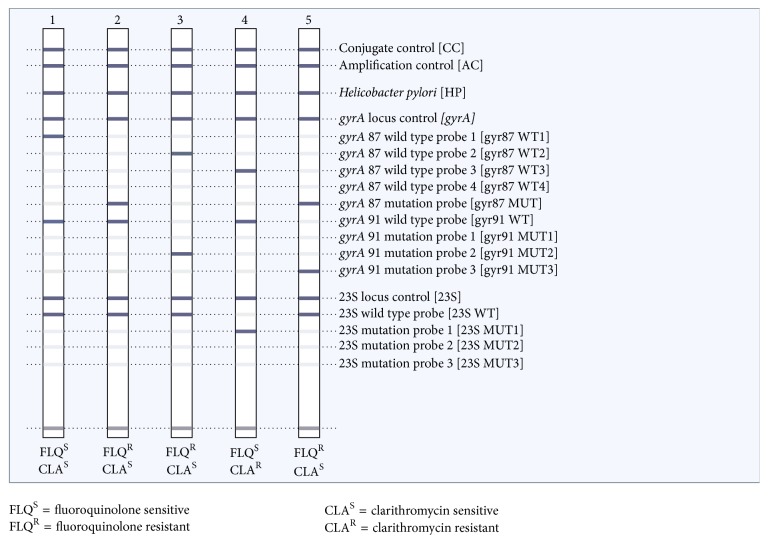
Prototype of the GTHDT result (downloaded from http://www.hain-lifescience.de/en/products/microbiology/helicobacter/genotype-helicodr.html).

*Quality Control and Interpretation of the Results*. During the entire experimental processes, measures were taken to avoid contamination and ensure validity of the results. Also, positive and negative controls were included at all steps from sample processing to hybridization. Data were cleaned before analysis. A result was considered valid if the* Helicobacter pylori* (HP), amplification control (AC), and conjugate control (CC) bands were all present. In addition, the locus controls (*gyrA*, 23S and RNA) must have stained positive when the HP zone indicated the presence of* H. pylori*. If neither the locus control probe nor the respective wild type or mutation probes of one of the two genes examined had developed, the result was considered invalid for interpretation of resistance to the respective drug. The bands formed were interpreted accordingly as shown in the prototype in Figure 1, and details are outlined in the GTHDT kit insert [21].

#### 2.5.3. Data Management

The collected data was entered into a Microsoft excel spread sheet and exported to SPSS software v.11 for the analysis. The proportions of positivity of each test and the proportion of* H. pylori* resistance to CLA and FLQ were computed using SPSS v.16 software.

## 3. Results

### 3.1. Baseline Characteristics of the Studied Participants

A total of 142 participants, 105 (74%) from Mulago, 22 (15%) from Mengo, and 15 (1%) from Case Medical Centre, were recruited. Of these, 79/142 (55.6%) were females. The participants were aged between 13 and 85 (mean age = 40, SD = 18). A total of 135 participants had had the symptoms of PUD for more than two weeks. In addition, at least 42% (59/142) admitted to have taken one or more antibiotic(s) during the course of illness. Furthermore, 114/142 (80%) had taken a drug within one week before the screening. Detailed data are shown in Table 1.

### 3.2. Taytec* H. pylori* Antigen Test Results

**Figure 2:**
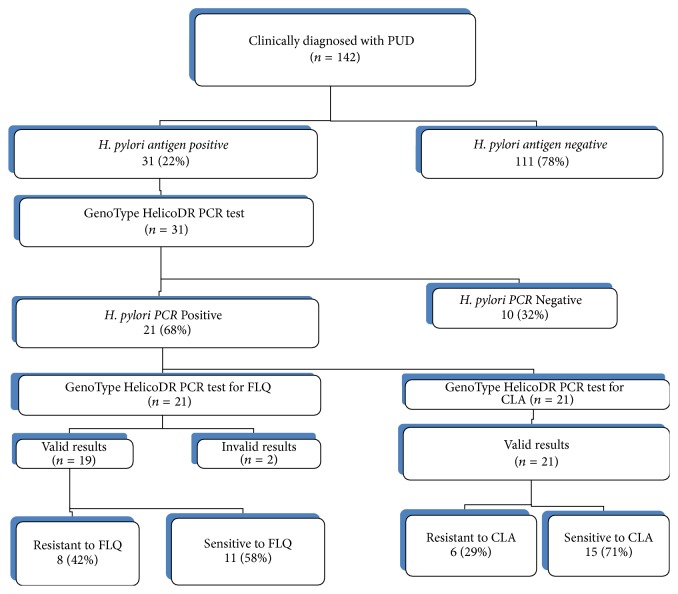
Study flowchart and summary of the results. Invalid results: lack of* H. pylori* control bands in the* gyrA* gene locus, CLA: clarithromycin, and FLQ: fluoroquinolones. Of the two FLQ invalid results, one was CLA susceptible and the other was resistant.

Out of the 142 stool samples, 31 were positive for the* H. pylori* antigen giving a prevalence of 22% in patients clinically diagnosed with PUD as shown in Figure 2.

### 3.3. GTHDT Results

In Figure 3 in the supplementary materials (available online at https://doi.org/10.1155/2017/5430723), we have shown representative results of hybridization on the nitrocellulose strips from our laboratory.

Of the 31* H. pylori* stool antigen positive samples, 21 (68%) tested positive with the GTHDT, as shown in Table 2 and Figure 3 in the supplementary materials. Among the 10 GTHDT negative samples, 7 were from patients who had taken antibiotics such as amoxicillin, metronidazole, and ceftrioxone, and one of these had also taken ciprofloxacin.

### 3.4. *H. pylori* Resistance to CLA and FLQ

#### 3.4.1. CLA

All the 21 samples which tested positive for* H. pylori* with the GTHDT gave valid CLA test results, and 6 (29%) were resistant to CLA as shown in Figure 3 in the supplementary materials.

#### 3.4.2. FLQ

Nineteen of the 21 antigen positive samples gave interpretable results for FLQ resistance testing, of which 8 (42%) were resistant as shown in Table 2 and Figure 2. Invalid results were due to lack of the* gyrA* gene locus control bands for* H. pylori*.

## 4. Discussion

In this study, we explored new simple to collect stool specimens as starting materials for use with the GTHDT for simultaneous detection for* H. pylori* and its resistance to CLA and FLQ. We also determined the proportions of* H. pylori* resistance to FLQ and CLA. We anticipate that our findings have the potential to inform clinicians on the level of* H. pylori* resistance to the selected antibiotics, which will help to improve on overall management of patients with suspected peptic or gastroduodenal ulcers. Additionally, with stool as specimen for this test, we anticipate high patient acceptability and reduce the costs to the patient/health systems of testing for* H. pylori*.

In this preliminary study only* H. pylori* antigen positive stool samples were subjected to the study test. Out of the* H. pylori* stool antigen positive specimens, the GTHDT showed a high positivity score of 68% (21/31). By study design, we did not aim at determining sensitivity or specificity of the GTHDT at this stage of new test evaluation, and another study to do this has been planned. However, the high positivity score obtained in this study provides strong evidence for stool as a possible specimen for same day molecular diagnosis of* H. pylori* and its resistance to CLA and FLQ. Our results are similar to those obtained in other studies on* H. pylori* diagnostics. In a study in 2016, Brennan et al. used the GenoType HelicoDR test to detect 80.3% (53/66) of* H. pylori* infection in stool samples [26]. In another study by Lottspeich et al., 63% of the confirmed stool samples turned positive for* H. pylori* when a real-time PCR assay was used [23]. Data from various studies indicate that antigen tests for* H. pylori* on stool samples have a high sensitivity and specificity [23]. The low performance of the PCR-based test in our study compared with the antigen test is disappointing but might be improved if antigen positive PCR-negative samples were retested, and/or the amount of stool/DNA used for the PCR-based test was increased as reported by Schabereiter-Gurtner et al. 2004 [27]. By repeating the stool antigen tests before PCR, Schabereiter-Gurtner and colleagues reported that the real-time PCR test was highly accurate in the detection of* H. pylori* infection in stool and allowed for culture-independent clarithromycin susceptibility testing [27]. Furthermore, in a systematic review and meta-analysis study in patients with bleeding peptic ulcer by Gisbert et al. in 2006 [28], the sensitivities of the different test methods were stool antigen test 87% (6 studies and 377 patients), rapid urease test 67% (16 studies and 1,417 patients), urea breath test 93% (8 studies and 520 patients), serology 88% (9 studies and 803 patients), histology 70% (10 studies and 827 patients), and culture 45% (3 studies and 314 patients). While our study did not perform formal computation of sensitivity of the GTHDT, the high positivity rate of 68% among the* H. pylori* antigen positive stool samples indicates this test to be technically promising and warranties well-designed prospective studies to determine the diagnostic accuracy of the GTHDT on stool specimens against an established gold standard. In our study, 32% of the hitherto* H. pylori* Ag positive stool samples remained negative at PCR. It is likely that nonspecific PCR inhibitors were responsible for this negativity. Optimization of DNA extraction methods or repeat testing as proposed by Makristathis and Hirschl may help to solve this false negativity problem but it should be noted that repeat testing is an expensive approach in routine patient care [29]. This could also be associated with mixed populations of the bacteria as reported by Schabereiter-Gurtner where it led to failure of PCR to detect the resistant genotype in the biopsy DNA, stool DNA, or both in one case [27].

### 4.1. Drug Resistant* H. pylori*

The 4th edition of the Maastricht consensus recommended a threshold of 15–20% to separate regions of high and low CLA resistance [30]. In our study,* H. pylori* resistance to CLA was high at 29%. Uganda can thus be considered an area of high CLA resistance. Our findings are in conformity with several global reports. The study by Lee et al. reported* H. pylori* resistance to CLA at 24.7% [12]. Another study by Kumala and Rani in 2006 found that* H. pylori* resistance to CLA was 28% [13]. In addition, CLA resistance in children is reported to be at 34% in Austria, 37% in France, 39% in Portugal, and 49.2% in Spain [31]. Our findings also agree with the 37% resistance to CLA reported in a Pakistan study [32], 20% in Southern European studies [33], 52% in Brazil [34], and 28% in Japan [35]. However, in USA, CLA resistance was found in only 8.6% [36, 37]. Some researchers have reported that a higher prevalence of resistance to both CLA and FLQ was detected in samples from stool compared to biopsy, and they suggested that this could be related to the existence of mixed infections with both resistant and susceptible strains [38]. Whereas this could partly explain our findings, it also could imply that when patients harbouring mixed infections receive antibiotics, the resistant strains naturally become selected for making the drug resistant problem even worse.

In Uganda clarithromycin is often used in first-line anti-PUD regimens, the so-called triple therapy without any susceptibility data or sometimes even a prescription. It is most likely that patients who tend to get recurrent disease might be harbouring super selected drug resistant* H. pylori.* Future studies need to focus on this area. In low clarithromycin resistance regions, treatment regimens containing clarithromycin are recommended as a first-line empirical treatment. In areas of high clarithromycin resistance such as in Uganda as from this study, first-line empirical treatment should utilize quadruple treatment regimens, that is, bismuth subsalicylate, proton pump inhibitors (PPI), tetracycline, and metronidazole [39]. After failure of the latter regimen, a fluoroquinolone-containing triple therapy should ideally be recommended [39].

The FLQ are usually considered safe oral antibiotics used as second-line drug for* H. pylori* infection treatment. The 42% resistance to FLQ in Uganda is not only very high but also worrying. In Uganda and many other developing countries with insufficient controls on sale of antibiotics, FLQ are used as first-line antibiotics for other diseases such as enteric upsets, cough, and urinary tract infections (UTIs). This could have led to super selection of the resistant strains and thus the relatively high resistance of* H. pylori* (42%) reported in the current study. Our findings are in agreement with previous studies where resistance to FLQ was found in 48%, 56%, and 62%, in Japan, China, and Pakistan, respectively [32, 40–42]. Furthermore, it is in agreement with the Lee et al. work carried out from 2003 to 2012 which reported an increased resistance rate of* H. pylori* to FLQ (levofloxacin and moxifloxacin) from 5% to 28% [12]. However, Hung et al. in their 2009 study in Taiwan found resistance in only 11% [43]. Additionally, Kumala and Rani study of 2006 also reported lower resistance of* H. pylori* to FLQ (ciprofloxacin, norfloxacin, and ofloxacin at 6.9%, sparfloxacin and gatifloxacin at 2.8%, and levofloxacin and moxifloxacin at 1.4%) [13]. In the developed countries, FLQ are reserved as second-line antibiotics for* H. pylori* treatment. In Africa, FLQ are first-line antibiotics for many other ailments increasing antibiotic resistance selection pressure, and it remains worrying what options will be there for the 42% patients with FLQ-resistant* H. pylori*.

## 5. Conclusions

This preliminary study has shown that stool is a possible specimen for use with the GTHDT in direct detection* of H. pylori* and its resistance to CLA/FLQ. We have further shown that* H. pylori* resistance to CLA and FLQ in Uganda is high. We recommend a prospectively designed study to determine the sensitivity and specificity of the GTHDT for diagnosis of* H. pylori* in stool samples against an established standard reference test and also determine the prevalence of* H. pylori* resistance to CLA and FLQ on larger sample sizes to guide patient care.

## 6. Limitations

The main limitation of our study is that we did not have resources for endoscopic biopsy and confirmatory diagnosis of* H. pylori* as we could not carry out the urease breath test, and we studied a biased population of stool antigen positive samples. However, we strongly believe that the evidence presented in this paper will ignite more studies to be conducted on this topic.

## Supplementary Material

Figure 3 (supplementary materials) shows representative hybridization results of the GTHDT on the nitrocellulose strips as obtained in our laboratory. In strip (a) the *H. pylori* strain was sensitive to both FQL and CLA because of presence of probe bands in the gyrA and 23S rRNA wild type loci but no mutation bands. In strip (b) the *H. pylori* strain was resistant to both FQL and CLA because of presence of mutation bands in both the gyrA 91 (MUT1) and 23S (MUT3) probe loci. In strip (c) the *H. pylori* strain was resistant to FQL because of presence of mutation band in the gyrA 91 (MUT1) but sensitive to CLA because of presence of the 23S wildtype probe bands but no mutation bands in the 23S (MUT1-MUT3) probe loci. In strip (d) (negative control) only the conjugate and amplification control bands are seen and no other bands because only PCR water was tested.
Kit insert for the Taytec® *Helicobacter pylori* antigen test in stool.

## Figures and Tables

**Table 1:** Baseline characteristics of studied patients (*N* = 142).

ITEM	Number (*n* = 142)	%
*Hospitals of recruitment*		
Mulago	105	74
Mengo	22	15
Case Medical Centre	15	1

*Gender*		
Female	79	56
Male	62	44
Not given	1	7

*Duration of illness*		
Up to 2 weeks	7	5
3-4 weeks	8	6
2 months to 1 year	40	28
2–5 years	61	43
More than 5 years	26	18

*Taken drug(s)*		
Yes	140	99
No	2	1

*The time the drug was last taken*		
Up to one week	114	83
Within a month	16	11
More than a month	10	7
NA	2	1

*Drug(s) taken*		
One antibiotic plus proton pump inhibitors (PPI)	41	29
Proton pump inhibitors (PPI) only	38	27
Triple therapy	28	20
Single antibiotic	19	13
Two antibiotics	12	9
Never taken peptic ulcer drugs	3	2
Herbal	1	0

*Specific antibiotics taken in last 2 weeks*		
FLQ taken		
No	135	95
Yes	7	5
CLA taken		
No	138	97
Yes	4	3
^*∗*^Other antibiotics taken		
Yes	94	66
No	48	34

CLA = clarithromycin, FLQ = fluoroquinolones. ^*∗*^Other antibiotics included amoxicillin, metronidazole, and ceftrioxone.

**Table 2:** Results of the Taytec *H. pylori* stool antigen and GTHDT (*N* = 31).

Serial number	Sex	Age (years)	Antibiotics taken within last two weeks			Taytec antigen test	GTHDT results		
			FLQ	CLA	^*∗*^Other antibiotics		*H. pylori*	CLA	FLQ
(1)	M	30	No	No	Yes	P	P	R	S
(2)	M	29	No	No	No	P	P	S	S
(3)	M	40	No	No	No	P	P	S	R
(4)	M	76	No	No	No	P	N	NA	NA
(5)	M	35	No	No	Yes	P	N	NA	NA
(6)	M	45	No	No	Yes	P	P	R	Invalid
(7)	M	25	No	No	No	P	P	S	S
(8)	F	58	No	No	Yes	P	N	NA	NA
(9)	F	23	No	No	No	P	P	S	S
(10)	F	85	No	No	Yes	P	P	S	S
(11)	M	20	Yes	No	Yes	P	N	NA	NA
(12)	F	80	No	No	Yes	P	P	S	S
(13)	F	16	No	No	Yes	P	N	NA	NA
(14)	F	44	No	No	Yes	P	P	R	R
(15)	F	32	No	No	No	P	P	S	S
(16)	F	75	No	No	No	P	N	NA	NA
(17)	M	57	No	No	Yes	P	P	S	R
(18)	F	55	No	No	Yes	P	P	S	S
(19)	M	24	No	No	No	P	P	R	R
(20)	F	35	No	No	Yes	P	N	NA	NA
(21)	F	42	No	No	Yes	P	P	S	R
(22)	F	43	No	No	No	P	P	R	R
(23)	M	17	No	No	No	P	P	S	S
(24)	M	33	No	No	No	P	P	S	Invalid
(25)	M	40	No	No	Yes	P	N	NA	NA
(26)	UNK	UNK	No	No	No	P	P	R	R
(27)	F	28	No	No	No	P	N	NA	NA
(28)	F	42	No	No	Yes	P	N	NA	NA
(29)	M	38	No	No	No	P	P	S	S
(30)	F	17	No	No	No	P	P	S	S
(31)	M	20	No	No	Yes	P	P	S	R

Total	M = 15		No = 30	No = 31	Yes = 16	P = 31	P = 21	R = 6	R = 8
								S = 15	S = 11
	F = 15		Yes = 1	Yes = 0	No = 15		N = 10	NA = 10	Invalid = 2
									NA = 10

CLA = clarithromycin, F = female, FLQ = fluoroquinolone, M = male, N = negative, NA = not tested, P = positive, S = sensitive, and UNK = unknown. ^*∗*^Other antibiotics included amoxicillin, metronidazole, and ceftrioxone.
