# Supplementary material for: Helicobacter pylori from Peptic Ulcer Patients in Uganda Is Highly Resistant to Clarithromycin and Fluoroquinolones: Results of the GenoType HelicoDR Test Directly Applied on Stool
Source: Biomed Res Int. 2017 May 7;2017:5430723. doi: 10.1155/2017/5430723 (PMC5438841; doi:10.1155/2017/5430723)
Supplement: Supplementary file 1 — Figure 3 (supplementary materials) shows representative hybridization results of the GTHDT on the nitrocellulose strips as obtained in our laboratory. In strip (a) the H. pylori strain was sensitive to both FQL and CLA because of presence of probe bands in the gyrA and 23S rRNA wild type loci but no mutation bands. In strip (b) the H. pylori strain was resistant to both FQL and CLA because of presence of mutation bands in both the gyrA 91 (MUT1) and 23S (MUT3) probe loci. In strip (c) the H. pylori strain was resistant to FQL because of presence of mutation band in the gyrA 91 (MUT1) but sensitive to CLA because of presence of the 23S wildtype probe bands but no mutation bands in the 23S (MUT1-MUT3) probe loci. In strip (d) (negative control) only the conjugate and amplification control bands are seen and no other bands because only PCR water was tested. Kit insert for the Taytec® Helicobacter pylori antigen test in stool. [file 5430723.f1.zip › 5430723.f1.pdf]

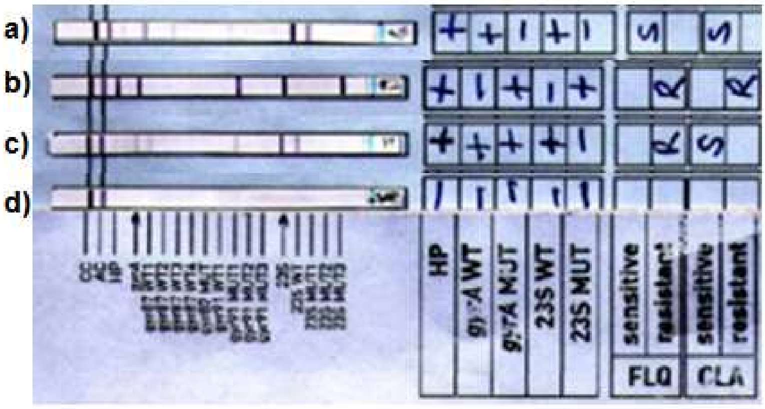

Figure 3. Representative hybridisation results on the nitrocellulose strips obtained in our laboratory.

a) *H. pylori* sensitive to both FQL and CLA, b) Resistant to both FLQ and CLA
c) Resistant to FLQ and but sensitive to CLA, d) Negative control (PCR water)
